# Concurrent Detection of Human Norovirus and Bacterial Pathogens in Water Samples from an Agricultural Region in Central California Coast

**DOI:** 10.3389/fmicb.2017.01560

**Published:** 2017-08-21

**Authors:** Peng Tian, David Yang, Lei Shan, Dapeng Wang, Qianqian Li, Lisa Gorski, Bertram G. Lee, Beatriz Quiñones, Michael B. Cooley

**Affiliations:** ^1^Produce Safety and Microbiology Research Unit, Western Regional Research Center, Agricultural Research Service, United States Department of Agriculture, Albany CA, United States; ^2^MOST-USDA Joint Research Center for Food Safety and Bor Luh Food Safety Center, School of Agriculture and Biology, Shanghai Jiao Tong University Shanghai, China; ^3^Department of Bioengineering, Shanghai Institute of Technology Shanghai, China

**Keywords:** human norovirus, bacterial pathogens, prevalence, watershed sites, agriculture, leafy green

## Abstract

Bacterial pathogens and human norovirus (HuNoV) are major cause for acute gastroenteritis caused by contaminated food and water. Public waterways can become contaminated from a variety of sources and flood after heavy rain events, leading to pathogen contamination of produce fields. We initiated a survey of several public watersheds in a major leafy green produce production region of the Central California Coast to determine the prevalence of HuNoV as well as bacterial pathogens. Moore swabs were used to collect environmental samples bi-monthly at over 30 sampling sites in the region. High prevalence of HuNoV and bacterial pathogens were detected in environmental water samples in the region. The overall detection rates of HuNoV, O157 Shiga toxin-producing *Escherichia coli* (STEC), non-O157 STEC, *Salmonella*, and *Listeria* were 25.58, 7.91, 9.42, 59.65, and 44.30%, respectively. The detection rates of *Salmonella* and *L. monocytogenes* were significantly higher in the spring. Fall and spring had elevated detection rates of O157 STEC. The overall detection rates of non-O157 STEC in the fall were lower than the other seasons but not significant. The overall detection rates of HuNoV were highest in fall, followed by spring and winter, with summer being lowest and significantly lower than other seasons. This study presented the first study of evaluating the correlation between the detection rate of HuNoV and the detection rates of four bacterial pathogens from environmental water. Overall, there was no significant difference in HuNoV detection rates between samples testing positive or negative for the four bacterial pathogens tested. Pathogens in animal-impacted and human-impacted areas were investigated. There were significant higher detection rates in animal-impacted areas than that of human-impacted areas for bacterial pathogens. However, there was no difference in HuNoV detection rates between these two areas. The overall detection levels of generic *E. coli* and detection rate of HuNoV showed no correlation.

## Introduction

Human noroviruses are highly contagious pathogens and the major cause for acute non-bacterial gastroenteritis. The stool of infected patients can contain high concentrations of virus (up to 10^11^ virus particles/g), and virus shedding can last up to 3 weeks (Atmar et al., 2008). In recent years, an increase in the number of bacterial pathogens as wells as human norovirus (HuNoV) outbreaks, which correlates with the increased consumption of minimally-processed (e.g., raw) fruits and vegetables has been observed (Lynch et al., 2009; Patel et al., 2009; Baert et al., 2011). It is possible that the produce was contaminated by bacterial pathogens and HuNoV via exposure to contaminated water from public waterways (rivers, lakes, ponds, streams, etc.). It has been reported that public waterways can provide a central reservoir for bacterial and enteric viral pathogens (Bosch, 1998; Hanning et al., 2009; Lynch et al., 2009; Kiulia et al., 2010; Oliveira et al., 2011). The water can become contaminated from a variety of sources, such as exposure to sewage, and agricultural runoff from livestock operations (Gagliardi and Karns, 2000; Walters et al., 2011). Public waterways can be contaminated by sewage containing enteric pathogens from the stool of infected patients. Wildlife is also suspected as a potential a vector for pathogen bacteria for water contamination (Kirk et al., 2002; Jay et al., 2007; Gorski et al., 2013). These contaminated waterways can flood and overflow after large rain events, which may lead to pathogen transmission into the fields.

It has been well-documented that HuNoV can be transmitted through contaminated water (Kukkula et al., 1997). Waterborne outbreaks have since been detected on the basis of epidemiologic evidence (Kaplan et al., 1982; Beller et al., 1997; Blackburn et al., 2004). Recently, several volumetric methods such as ultracentrifugation, polyethylene glycol (PEG) precipitation, and membrane-based filtration/elution methods (Katayama et al., 2002; Gentry et al., 2009b) have been developed and proven to be effective methods to recover enteric viruses from environmental water samples. Aw et al. (2009) reported on the prevalence and genotypes of HuNoV in the tropical surface waters of urban Singapore finding dominant HuNoV genotypes to be GI.2, GI.4, and GII.4. A waterborne HuNoV outbreak at New Zealand has been reported (Hewitt et al., 2007). HuNoV GI.5 was identified in water samples and linked to clinical fecal specimens, providing clear evidence of the predominant pathogen and route of exposure. HuNoV could be detected in 23.0 and 33.9% of environmental water samples collected from southern Brazil (Victoria et al., 2010) and from northern Brazil (Teixeira et al., 2017), respectively. Love et al. (2014) reported that HuNoV could be detected in 22.3% of marine water samples collected from two recreational beaches in southern California. HuNoV could be detected in 8.3% estuarine water samples collected in Sapelo Sound and Wassaw Sound in Georgia (Gentry et al., 2009a). HuNoV could be detected in 10.0% of samples collected from the Milwaukee River watershed for GI and 1.6% for GII (Corsi et al., 2014). HuNoV GI, GII and bacterial pathogens could be detected in 2.4, 1.0, and 1.4% respectively of water samples collected in Great Lakes tributaries, United States (Lenaker et al., 2017). However, HuNoV prevalence in public watersheds near a major leafy green produce production region has never been reported.

Previously, we have reported the prevalence of Shiga-toxin-producing *Escherichia coli* (STEC), *Salmonella*, and *Listeria* in public watersheds in a major leafy green produce production region of Central Coastal California (Cooley et al., 2014). However, the prevalence of HuNoV in the area has not been reported. The objective of this study was to determine prevalence of HuNoV in this area and to determine the correlation between the prevalence of HuNoV and the bacterial pathogens. The present report consists of pathogen-prevalence data for HuNoV and four bacterial foodborne pathogens (STEC, non-O157 STEC, *Salmonella*, and *Listeria*) at 30 different sites representing five watersheds in a major produce production region over the course of 2 years.

## Materials and Methods

### Sampling Locations

**FIGURE 1:**
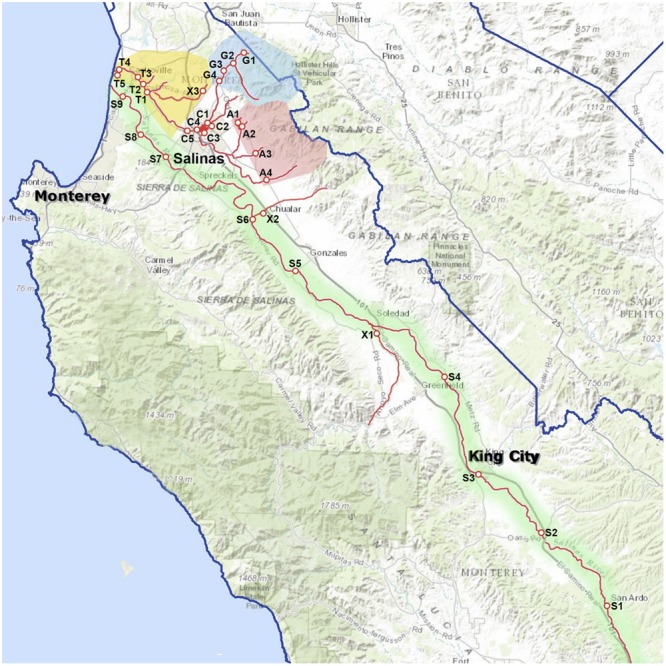
Map of the Salinas Valley, sampling sites, and groupings by region: Tembladero Slough region = sites T1 through T5; Carr Lake region = sites C1 through C5; Gabilan Creek region = sites G1 through G4; Alisal Creek region = sites A1 through A4; Upper Salinas River region = sites S1 through S5; Lower Salinas River region = sites S5 through S9; X = site X1-X3, sampled regularly but do not fit into any of the designated watersheds.

Sampling sites in Monterey County in California, United States were selected on the basis of accessibility and previous experience with the watersheds gained from a collaboration with the Central Coast Water Quality Control Board. Sampling sites are shown in **Figure 1**. Most sites were grouped into seven regions by watershed when possible: Tembladero Slough (T1 through T5), Carr Lake (C1 through C5), Gabilan Creek (G1 through G4), Alisan Creek (A1 through A4), upper Salinas River (S1 through S5), lower Salinas River (S6 through S9), and the regions that do not fit into any of the designated watersheds (X1 through X3). It needs to be noted that the Tembladero Slough and Carr Lake regions are downstream and within the city of Salinas (respectively), and are potentially impacted by seepage from septic systems. The lower Salinas River flows through eight small towns before ending at Monterey Bay near the city of Salinas and north of the city of Monterey. Therefore, these regions (Tembladero Slough, Carr Lake, and Lower Salinas River) and their sampling sites were regarded as human-impacted. The other regions were surrounded by ranches on both sides of the valley, with varying densities of grazing cattle and wild animals. These regions were considered animal-impacted as they were exposed to some extent by wildlife in riparian areas, and runoff from both agriculture and cattle ranches (primarily cow-calf operations) at various times of the year.

### Sampling Method

**FIGURE 2:**
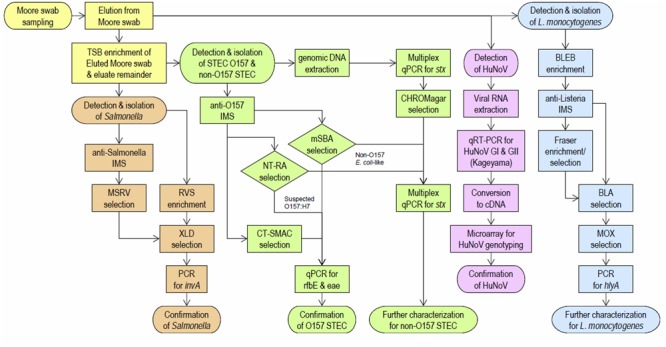
Overview of the Moore swab workflow.

At approximately 2-week intervals over a 2-year period, Moore swabs (cut cotton gauze gathered and tethered with a string) were deployed for 24 h in the lakes, rivers, streams, and ponds of selected sampling sites in Monterey County. Samples were not obtained from some sample sites at certain sampling intervals due to reasons such swab loss (i.e., missing from where it was originally deployed, the loss discovered upon return for collection), or simply the absence of water at the sampling site. The Moore swab workflow is summarized in **Figure 2**. Briefly, collected swabs were placed into Whirl-Pak bags, kept on ice, transported to the lab and processed immediately. Five hundred milliliters of sterile water was added to each bag, followed by vigorous shaking by hand for 20 s. From the swab eluate, 100 ml was portioned for *L. monocytogenes* isolation, 50 ml was portioned for norovirus analysis, and 100 ml was portioned for generic *E. coli* and other research not described here. To the remaining swab and eluate in the Whirl-Pak bag, 30 ml of 10x Tryptic Soy Broth (TSB) was added, and the bag was incubated at 25°C for 2 h, then 42°C for 8 h. The TSB-enriched cultures were used for STEC and *Salmonella* isolations.

### Detection and Isolation of O157 STEC and Non-O157 STEC

O157 STEC and non-O157 STEC were isolated by methods published previously (Cooley et al., 2013). Briefly, genomic DNA was heat-released from TSB-enriched culture and measured by qPCR using a multiplexed primer set for all shiga toxin (stx) types (Cooley et al., 2013). The RT-qPCR-positive TSB-enriched cultures were streaked onto CHROMagar O157 media plates (DRG International, Mountainside, NJ, United States) and single, mauve, *E. coli-*like colonies were selected for a second round of qPCR quantitation using the *stx* multiplex primer set. In a parallel procedure, TSB-enriched culture was subjected to Immuno Magnetic Separation (IMS) with anti-O157 antibody (Invitrogen/Dynal, Carlsbad, CA, United States), and the collected and re-suspended IMS beads were spread on three types of media as described previously (Cooley et al., 2013). All plates were incubated at 37°C for 24 h. Suspected O157:H7 colonies were selected on the basis of colony color, and were analyzed by qPCR for the presence of the O157 O-antigen synthesis (*rfb*E) and intimin (*eae*) genes (Cooley et al., 2007). Additionally, non-O157 *E. coli*-like colonies were selected from NT-RA (red colonies) and mSBA (blue colonies that showed hemolytic activity) and analyzed by qPCR using the *stx* multiplex primer set described above.

### Detection and Isolation of *Salmonella*

The same enriched culture used for the detection and isolation of STEC were also used for *Salmonella*. Detection and isolation of *Salmonella* was performed in two parallel procedures by prior-reported methods (Cooley et al., 2007; Kalchayanand et al., 2009; Gorski et al., 2011, 2013). For the first method, portions of each TSB-enriched culture was subject to IMS with anti-*Salmonella* antibody (Dynal, Invitrogen), then the collected and re-suspended beads were inoculated into Rappaport-Vasilliadis Soya Peptone Broth (RVS, Oxoid, Remel, Inc., Lenexa, KS, United States) and incubated. The RVS culture was streaked onto Xylose Desoxycholate Agar (XLD, Difco, Becton Dickinson-BBL, Franklin Lakes, NJ, United States). For the second method, portions of each TSB-enriched culture were plated onto Modified Semi-solid Rappaport Vasilliadis (MSRV) medium and resulting motile growth was picked and streaked onto XLD. In both methods, colonies that were black on XLD were picked and were confirmed as *Salmonella* by PCR for the *invA* gene (Gorski et al., 2011, 2013).

### Detection of and Isolation of *Listeria monocytogenes*

Enrichment, isolation and detection of *L. monocytogenes* was performed as described previously (Cooley et al., 2014). Swab eluate aliquots were enriched with concentrated Buffered *Listeria* Enrichment Broth Base (BLEB, Difco) for 18 h at 30°C. The BLEB-enriched cultures were subjected to IMS with anti-*Listeria* antibody (Dynal, Invitrogen), and two parallel methods were used to detect *L. monocytogenes* from the collected and re-suspended beads. For the first method, aliquots of re-suspended beads were inoculated into Fraser Broth and incubated at 37°C. Fraser Broth cultures that exhibited esculin hydrolysis (indicated by blackened media) were streaked onto Brilliance *Listeria* Agar plate (Oxoid, Remel, Lenexa, KS, United States). For the second method, aliquots of the re-suspended beads were plated directly onto Brilliance *Listeria* Agar. All Brilliance *Listeria* Agar plates were incubated for 2 days at 37°C, and blue colonies surrounded by clearing of the surrounding media were picked and streaked onto Modified Oxford Agar (MOX). Bluish-white colonies from MOX were selected for detection of the *hlyA* gene by PCR (Cooley et al., 2014).

### Quantitation of Generic *E. coli*

One hundred milliliters of swab eluate was incubated at 37°C for 24 h in separate and sealed QuantiTray 2000 with Colilert reagent (IDEXX Laboratories, Westbrook, Maine). Swab eluates were also diluted 100-fold and cultured in separate Colilert trays in order to obtain counts greater than 2419 most probable number (MPN) per 100 ml (Benjamin et al., 2013). The MPN of *E. coli* per 100 ml or per swab was determined by fluorescent well count according to manufacturer’s protocol.

### Process Control Virus (PCV) for Environmental Water Samples

Tulane virus (TV) was spiked into each environmental water sample to evaluate efficacy of viral RNA extraction and the presence of RT-PCR inhibitors. Briefly, 10 μl of TV (2 × 10^6^ viral genomic copies) was spiked into each swab eluate. The viral RNA was extracted from swab eluate using a QIAamp RNA Viral Mini extraction kit (Qiagen United States, Valencia, CA, United States) with a high-throughput QIAvac 24 Plus manifold (Qiagen United States, Valencia, CA, United States) in accordance with the kit manufacturer’s manifold extraction protocol. The primers and probes used for detection of TV were: TV forward (5′-TGA CGA TGA CCT TGC GTG-3′), TV reverse (5′-TGG GAT TCA ACC ATG ATA CAG TC-3′), TV probe (5′-HEX- ACC CCA AAG CCC CAG AGT TGA T -BHQ-1-3′) (Tian et al., 2013). Each 25 μl reaction consisted of 12.5 μl of Quantitect Probe RT-PCR master mix, 7.5 μl of RNAse-free water, 0.75 μl of each primer (TV forward, TV reverse, both at 10 μM), 0.25 μl of TV probe at 10 μM, 0.25 μl of Quantitect RT mix, and 3 μl of extracted RNA. Cycling times and temperatures were 50°C for 30 min, 95°C for 15 min, followed by 45 cycles of 95°C for 15 s, 53°C for 20 s, and 60°C for 50 s. Fluorescence was read at the end of each 60°C extension step. Data acquisition was conducted by MxPro (Stratagene; La Jolla, CA, United States), with threshold determination at default, automated settings. Both undiluted and 1:10 diluted RNA extractions were used for detection of TV.

### Detection of HuNoV

Putative viral RNA extract was screened for HuNoV GI and GII amplicon signals with a probe-based, multiplexed quantitative real-time RT-PCR using a commercial one-step RT-qPCR kit (Quantitect Probe RT-PCR kit, Qiagen United States; Valencia, CA, United States) with a half-scale modification of the manufacturer’s protocol. The Kageyama-designed primers and probes were used for detection of GI and GII HuNoV (Kageyama et al., 2003). Each half-scale 25 μl reaction consisted of 12.5 μl of Quantitect Probe RT-PCR master mix, 5.5 μl of RNase-free water, 0.75 μl of each 10 μM primer (COGIF and COGIR; COGIIF and COGIIR), 0.25 μl of each 10 μM probe (GI-P1 6-FAM, GI-P1b-1 6-FAM, and GII-P HEX), 0.25 μl of Quantitect RT mix and 3 μl of putative viral RNA extract. Cycling times and temperatures were 50°C for 30 min, 95°C for 15 min, followed by 45 cycles of 95°C for 15 s, 53°C for 20 s, and 60°C for 50 s. Fluorescence was read at the end of each 60°C extension step. Automated thermal cycling and data acquisition was performed on a MX3000P qPCR system and MxPro software (Stratagene; La Jolla, CA, United States), with threshold determination at default settings. For each Ct-generating sample, the overall shape of the amplification curve and the slope of the linear phase (if identifiable) were compared against those of the HuNoV RNA positive control extracted from stool samples (GI.1 and GII.4) in order to eliminate false-positive Ct values arising from artifacts of automated threshold determination, and grossly inefficient amplification (presumed to be due to mis-priming). Both undiluted and 1:10 diluted RNA extractions were used for detection of HuNoV. Results were presented as positive or negative as the variance of PCR inhibitors between samples made quantitative analysis impossible. For the genotyping of HuNoV strains within a genogroup, RNA samples were subjected to RT-PCR with OneStep RT-PCR kit (Qiagen) using either G1SKF/R or G2SKF/R primers (Kojima et al., 2002). The amplified products were further purified with MinElute purification kit (Qiagen) and subjected to DNA sequencing by Elim Biopharmaceuticals, Inc. (Hayward, CA, United States). The detection rate for a norovirus genotype was determined by dividing the number of Moore swab elutes that tested positive for a particular norovirus genotype by the 860 total Moore swab elutes.

### Statistical Analysis

Comparisons of detection rates were analyzed by Chi-square using Sigma Stat version 3.0 (SPSS, Chicago, IL, United States). A *p*-value of less than 0.05 was considered significant.

## Results

### Evaluation of TV Used as a PCV for Environmental Water Samples

In a pilot experiment, 250 environmental samples were spiked with TV for use as an internal control to indicate and facilitate the quantitation of RT-qPCR inhibition. No inhibition of spiked TV virus was observed in 133 samples with the expected magnitude of the TV genomic signals. Partial TV-inhibition was observed in 34 samples with an average increase in Ct value by at least four units. Complete-TV-inhibition was observed in 83 samples with complete absence of the otherwise-expected TV genomic signal. From these 250 samples, 44 and 16 were observed with GI and GII HuNoV genomic signals, respectively. Although the detection rates of GI or GII HuNoV were higher in samples without observed TV signal inhibition (24.8% for GI and 8.3% for GII) than in those with partial TV- inhibition (20.6% for GI and 5.9% for GII), and complete TV- inhibition (16.9% for GI and 3.6% for GII) samples, the differences were not statistically significant (*p* > 0.05). In addition, there was no significant difference in Ct values for HuNoV in samples with or without TV-inhibition. The average Ct values for GI HuNoV in samples without observed inhibition, partial inhibition, and complete inhibition were 35.41 ± 2.85, 36.57 ± 1.82, and 34.75 ± 2.72, respectively. The average Ct values for GII HuNoV in samples without observed inhibition, partial inhibition, and complete inhibition were 31.71 ± 1.52, 32.69 ± 1.87, and 30.30 ± 3.08, respectively. The RNA extracts from the 83 TV-complete inhibition samples were further 10-fold diluted and retested by RT-qPCR for both TV and HuNoVs. Only nine samples became positive for TV after re-tested at 10-fold dilution and the other 74 samples remained undetectable for TV indicating limited effect of dilution samples on removal of inhibitors for TV. However, 28 more GI and none GII HuNoV were detected from these samples. We noticed that the inhibitory effect upon the amplification of TV and HuNoV genomic signals were different. There was no good correlation between inhibition of TV and HuNoV. More HuNoV could be detected when samples were diluted regardless of the inhibition status of the spiked TV. Therefore, TV was not tested in later samples. Samples were tested at undiluted and 1:10 diluted to reduce effect of RT-PCR inhibitors. Samples were not further diluted beyond 1:10 and re-tested as the expected Ct values were below the detection limits.

### Detection Rates of HuNoV and Bacterial Pathogens

Overall, out of 860 swab eluates tested, 221 (25.7%) tested positive for HuNoV, 68 (7.9%) tested positive for O157 STEC, 513 (59.7%) tested positive for *Salmonella*, 381 (44.3%) tested positive for *L. monocytogenes* and 81 (9.4%) tested positive for non-O157 STEC (**Table 1**). From 860 samples, 173 tested positive for HuNoV GI (20.1%), 102 tested positive for HuNoV GII (11.9%), 54 tested positive for both (6.3%), and 221 tested positive for either GI or GII (25.7%). Overall, HuNoV GI detection rate was higher than HuNoV GII detection rate (*p* < 0.00001). The detection rate for GI.1, GI.2, GI.3, GI.4, and GI.6 was 2.8, 1.1, 1.3, 29.0, 4.1%, respectively. The detection rate for GII.1, GII.2, GII.4, and GII.12 was 0.6, 0.1, 1.0, 7.8%, respectively. GI.4 and GII.12 were predominant genotypes for GI and GII, respectively.

**Table 1:** Seasonal detection rates of bacterial pathogens and HuNoV.

Season	HuNoV			Bacterial pathogens						
	GI	GII	GI and GII	GI or GII	O157	*Listeria*	*Salmonella*	Non-O157	*E. coli* (MPN)	*SD*
Spring	22.07%	11.73%	4.19%	29.61% b	9.50% b	56.43% b	70.67% b	10.34% a	2.05E+05	7.20E+05
Summer	12.05%	3.57%	2.23%	12.95% a	4.02% a	32.59% a	49.55% a	9.82% a	6.35E+05	7.21E+06
Fall	41.18%	9.41%	9.41%	41.18% c	11.75% b	32.94% a	48.24% a	5.88% a	1.43E+05	4.80E+05
Winter	17.10%	22.80%	13.47%	26.43% b	7.77% ab	40.42% a	55.96% a	8.81% a	7.74E+05	6.44E+05
All	20.11%	11.86%	6.28%	25.70%	7.91%	44.30%	59.65%	9.42%	1.62E+05	6.13E+05

*Shared letters in the column indicated no statistical difference among groups (*p* > 0.05). Different letters indicated there was a statistical difference (*p* < 0.05) between groups.*

### Seasonal Distribution of HuNoV and Bacterial Pathogens

The overall detection rate for HuNoV was highest in fall (41.2%), followed by spring (29.6%), winter (26.4%), and summer (13.0%) (**Table 1**). The overall detection rate for HuNoV was significantly lower (*p* < 0.00001) in summer relative to the other seasons. There was no significant difference (*p* = 0.81) in HuNoV detection rates among other seasons. Seasonal differences in the overall detection rates of bacterial pathogens were also identified. The overall detection rates for O157 STEC in summer was significantly lower than that of in fall and spring (*p* < 0.05). The detection rates for *Salmonella* and *Listeria* in spring were significantly higher than other seasons (*p* < 0.05). The detection rates for non-O157 STEC were similar in all seasons. Although highest level in summer and lowest level in winter were found, the detection levels for generic *E. coli* were not significantly different among all seasons due to huge variations (*p* > 0.05).

### Correlation between Detection of HuNoV and Bacterial Pathogens

The detection rates for HuNoV were compared between samples testing positive and negative for bacterial pathogens (**Table 2**). Overall, there was no difference in the detection rates for HuNoV between samples testing positive and negative for bacterial pathogens. Among 68 samples testing positive for O157 STEC, the HuNoV detection rate was 26.5%, which was not significantly different from the HuNoV detection rate of 25.6% in 792 samples testing negative for O157 STEC (*p* = 0.88). Likewise, among 221 samples testing positive for HuNoV, the O157 STEC detection rate was 8.1%, which was not significantly different from the O157 STEC detection rate of 7.8% in 639 samples testing negative for HuNoV. Among 381 samples testing positive for *L. monocytogenes*, the HuNoV detection rate was 25.2%, which was not significantly different (*p* = 0.76) from the HuNoV detection rate of 26.1% in 479 samples testing negative for *L. monocytogenes*. Likewise, among 221 samples testing positive for HuNoV, the *L. monocytogenes* detection rate was 43.4%, which was not significantly different from the *L. monocytogenes* detection rate of 44.6% in samples testing negative for HuNoV. For *Salmonella* and non-O157 STEC, there were no significant differences (*p* = 0.51 and *p* = 0.75) in HuNoV detection rates between samples testing positive and negative for either bacterial pathogen. Likewise, there were no significant differences in either of these two bacterial detection rates between samples testing positive and negative for HuNoV. For generic *E. coli*, there was no significant difference (*p* = 0.11) in detection rates between samples testing positive for HuNoV (1.78 × 10^5^) and samples testing negative for HuNoV (1.56 × 10^5^).

**Table 2:** Correlation between HuNoV and bacterial pathogens.

	HuNoV+	HuNoV-	Total	HuNoV%
O157+	18	50	68	26.47
O157-	203	589	792	25.63
Total	221	639	860	25.70
O157%	8.14%	7.82%	7.91%	*p* = 0.88
Lis.+	96	285	381	25.20
Lis.-	125	354	479	26.10
Total	221	639	860	25.70
Lis.%	43.44%	44.60%	44.30%	*p* = 0.76
Sal.+	136	377	513	26.51
Sal.-	85	262	347	24.50
Total	221	639	860	25.70
Sal.%	61.54%	59.00%	59.65%	*p* = 0.51
Non-O157+	22	59	81	27.16
Non-O157-	199	580	779	25.55
Total	221	639	860	25.70
Non-O157%	9.95%	9.23%	9.42%	*p* = 0.75
*E. coli*	1.78E+05	1.56E+05		*p* = 0.11

*O157, Lis., Sal., Non-O157, and HuNoV were abbreviations for STEC O157, *L. monocytogenes, Salmonella*, non-O157 STEC and human norovirus, respectively.*

### Detection of Bacterial Pathogens and HuNoV at Potential Human-Impacted and Animal-Impacted Areas

There were significant higher detection rates of bacterial pathogens in animal-impacted areas than human-impacted areas. The detection rates for O157 STEC, *L. monocytogenes, Salmonella* and non-O157 STEC in animal-impacted areas were 10.1, 49.3, 74.1, and 16.5% which were significantly higher than that of in human-impacted areas (**Tables 3A,B**). However, the detection rates for human norovirus were similar in human-impacted and animal-impacted areas (*p* = 0.095). There were some hot spots for bacterial pathogens (**Table 3C**). The detection rates for O157 STEC and *L. monocytogenes* within the Gabilan Creek region were significantly higher than that of other regions (*p* < 0.0005 and *p* < 0.025, respectively). The detection rates of *Salmonella* within the Gabilan Creek region was also significantly higher than that of other groups except upper Salinas River (*p* < 0.015). The detection rates of non-O157 STEC within the Gabilan Creek region and upper Salinas River were significantly higher than (*p* < 0.05) the other locations. The detection rates for HuNoV were similar among different regions (*p* > 0.05) except for Carr Lake (C) which was lower than the other regions. There was no correlation between HuNoV detection rates and the level of MPN of *E. coli*. Upper Salinas River had the lowest MPN but relative high detection rate of HuNoV.

**Table 3:** Detection of bacterial pathogens and HuNoV at potential human-impacted **(A)** and animal-impacted areas **(B)** as well as different regions **(C)**.

	O157	*Listeria*	*Salmonella*	Non-O157	HuNoV	*E. coli* (MPN)	*SD*	Total number
**(A)**								
S6	0	7	14	3	6	5.29E+04	1.00E+05	22
S7	0	13	19	0	7	9.78E+04	3.09E+05	36
S8	0	9	21	1	11	2.25E+04	6.67E+04	35
S9	0	2	5	1	7	6.55E+03	1.27E+04	32
C1	2	7	8	3	5	9.50E+04	1.51E+05	23
C2	0	13	13	0	4	1.55E+05	4.56E+05	24
C3	4	15	22	2	8	5.01E+05	1.16E+06	37
C4	3	14	17	1	5	1.34E+05	3.76E+05	36
C5	2	14	20	0	5	2.58E+05	9.17E+05	36
T1	5	16	13	0	8	3.00E+05	1.08E+06	36
T2	1	19	17	0	6	8.34E+04	1.43E+05	34
T3	4	18	17	2	10	1.32E+05	4.23E+05	30
T4	5	21	17	1	13	5.87E+04	1.38E+05	37
T5	1	13	9	0	11	3.42E+04	7.84E+04	36
Total	27 (5.95%)	181 (39.87%)	212 (46.70%)	14 (3.08%)	106 (23.35%)	1.38E+05	1.34E+05	454

	**O157**	***Listeria***	***Salmonella***	**Non-O157**	**HuNoV**	***E. coli* (MPN)**	***SD***	**Total number**

**(B)**								
S1	0	4	33	11	7	3.09E+04	6.99E+04	36
S2	0	2	30	7	8	4.53E+04	1.65E+05	37
S3	1	10	19	6	7	3.94E+04	5.03E+04	24
S4	4	10	19	8	8	6.40E+04	8.72E+04	24
S5	0	14	20	3	9	2.96E+04	2.03E+04	23
G1	5	15	17	6	10	5.12E+05	1.40E+06	28
G2	11	37	31	10	12	3.88E+05	9.34E+05	38
G3	11	23	22	4	5	2.94E+05	3.68E+05	25
G4	4	12	15	3	4	1.07E+05	1.90E+05	18
A1	2	0	0	1	0	2.52E+05	4.98E+05	4
A2	0	2	1	4	2	1.47E+05	3.36E+05	7
A2^∗^	2	9	8	0	1	5.35E+04	5.03E+04	12
A3	0	7	6	2	8	7.50E+04	1.43E+05	19
A4	0	13	30	0	12	1.74E+05	4.53E+05	36
X1	0	1	1	0	1	1.60E+03	Only 1 sample	1
X2	0	16	20	1	15	3.14E+04	7.93E+04	38
X3	1	25	29	1	6	4.96E+05	1.33E+06	46
Total	41 (10.10%)^∗^	200 (49.26%)^∗^	301 (74.14%)^∗^	67 (16.50%)^∗^	115 (28.33%)	1.61E+05	1.63E+05	406
	*p* = 0.024	*p* = 0.0056	*p* = 0.00	*p* = 0.00	*p* = 0.0954	*p* = 0.984		

*^∗^ Represented that there was statistical difference (*p* < 0.05) in detection rates between the human-impact area ***(A)*** and the animal-impact area ***(B)***.*								

	**O157**	***Listeria***	***Salmonella***	**Non-O157**	**HuNoV**	***E. coli* (MPN)**	***SD***

**(C)**							
upper	3.47% b	27.78% ab	84.03% c	24.31% c	27.08% bc	4.18E+04	1.40E+04
Lower	0% a	24.8% a	47.2% a	4% ab	24.8% ab	4.49E+04	3.48E+04
G	28.44% d	79.82% e	77.98% bc	21.10% c	28.44% bc	3.25E+05	1.70E+05
C	7.05% bc	40.39% c	51.28% a	3.85% ab	17.31% a	2.29E+05	1.64E+05
A	5.13% bc	39.74% bc	38.46% a	8.97% b	29.49% bc	1.40E+05	7.97E+04
T	9.25% c	50.29% dc	42.2% a	1.73% a	27.75% bc	1.22E+05	1.06E+05
X	1.33% ab	56% d	66.67% b	2.67% ab	29.33% bc	1.76E+05	2.77E+05
Average	7.91%	44.30%	59.65%	9.42%	25.58%	1.62E+05	6.13E+05

*Shared letters in the column indicated no statistical difference among groups (*p* > 0.05). There was statistical difference (*p* < 0.05) between groups had different letters.*

## Discussion

The present report documents a survey of pathogen prevalence within the watersheds of the Salinas Valley, a region that produces *>*60% of the leafy greens for the United States. The five watersheds of the Salinas Valley were represented by 30 publicly-accessible sampling sites for environmental water. While some of the data for bacterial pathogens was analyzed and published in a separate paper (Cooley et al., 2014), this report presents an integrated analysis of HuNoV and bacterial pathogen detection rates, and focuses on correlations between the detection rates of HuNoV and bacterial pathogens. We believe this is the first study of this nature conducted in an agricultural region for leafy greens.

Although several volumetric methods such as ultracentrifugation, PEG precipitation, and membrane-based filtration/elution methods (Katayama et al., 2002; Gentry et al., 2009b) have been developed and widely used in testing viruses in environmental water samples, these methods were not applicable in this study. For this study, the collection of large volumes of environmental water samples necessary to produce detectable signal for pathogens at low titers was either impractical or impossible at most collection sites. Therefore, the Moore swab technique was employed to sample environmental waters for 24 h to increase the odds of capturing and signal strength for low titer bacterial and viral pathogens. The Moore swab has been used for sampling environmental waters for bacterial pathogens (Cooley et al., 2007, 2014) and enteric viruses (Sattar and Westwood, 1977).

Mengovirus, Murine norovirus (MNV-1), MS2, Tulane virus (TV), Adenovirus 5 (Ad5), and turnip crinkle virus (TCV) have been used as PCVs for detecting enteric viruses from food and environmental samples (Hata et al., 2011; Gentry-Shields and Jaykus, 2015; Hennechart-Collette et al., 2015). Hennechart-Collette et al. (2015) demonstrated that MNV-1 was a good PCV for validating the detection of HAV and HuNoV GII in three food matrices tested and Mengovirus was a good PCV for HuNoV GI in bottle waters and semi-dried tomatoes. Hata et al. (2011) demonstrated that nucleic acid loss during the extraction process attributed to underestimation of the process of viral genomes in the environmental water samples when Ad5 and MNV-1 were used as PCVs. Gentry-Shields compared Mengovirus, MNV-1, MS2, TV and TCV as PCVs for use in extraction and detection of HuNoV from various food matrices (Gentry-Shields and Jaykus, 2015). They demonstrated that all PCVs did not behave equivalently and suggested that PCV should be used on an application-by-application basis. In this study, we attempted to use TV as a PCV for environmental water samples. We found that complete inhibition of TV genomic signal was observed in over 30% of samples tested. It is likely that false negative results were caused by the presence of inhibitors for RT-qPCR as dilution of TV-negative samples tested positive for both TV and HuNoV in some cases. However, there was no good correlation between inhibition of TV and inhibition of HuNoV measured by RT-qPCR. First, HuNoV could be detected at similar rates regardless of the presence or absence of the TV genomic signal. Second, 10-fold dilution of extracted sample RNA had a more limited effect on recovery of otherwise-expected TV genomic signal than that of HuNoV, as only 11% of expected TV genomic signal was recovered, while more significant recovery of HuNoV genomic signal (33.7%) was observed. Since inhibitors such as polysaccharides, humic acids, tannic acids, fulvic acids and terpenoids could either inhibit the activity of the polymerase or directly bind to nucleic acids and prevent amplification, it is possible that inhibition was sequence specific. Finally, false negatives may be present, as dilution of samples could reduce viral genomic titers below the detection limit. Therefore, a better internal control is needed to actually reflect the inhibitor status as well as processing status for HuNoV in the environmental waters if RT-qPCR method is used.

The seasonal distributions of bacterial and viral pathogens were different. The detection rates for non-O157 STEC were similar in all seasons. The detection rates for *Salmonella* and *Listeria* in spring were significantly higher than other seasons. The overall detection rates for O157 STEC in summer was significantly lower than that of in fall and spring. The overall detection rate for HuNoV was also significantly lower in summer relative to the other seasons. This may be a result of the increased rates of viral degradation from higher temperatures and stronger UV irradiation in the warmer, sunnier seasons.

In this study, we calculated for correlations between the detection rates of HuNoV and those of bacterial pathogens detection of the other four bacterial pathogens. The detection rates for HuNoV were similar in each bacterial pathogens positive and negative samples. The detection rates for each bacterial pathogens were also similar in both HuNoV-positive and HuNoV-negative samples. As far as we know this was the first study to demonstrate that there was no significant correlation between the detection rates of bacterial pathogens and HuNoV.

The presence and level of coliforms is used as an indicator for fecal contamination of water. Generic *E. coli* is a normal part of the gastrointestinal microbiota of humans and warm-blooded animals, exhibiting broad thermal tolerance, and is therefore found in human and animal feces. Many disease-causing pathogens are transferred from human and animal feces to water, and cause disease if they are ingested by people through contaminated water or water-irrigated produce. Generic *E. coli* has been used as an indicator of fecal contamination since some pathogens, notably viruses such as HuNoV, are difficult (or impossible) to culture and/or test directly. Pusch et al. (2005) reported that high level of enteric viral pathogens were detected in environmental waters samples where microbiological parameters such as generic *E. coli* and coliphages were at acceptable levels. Lopez-Galvez et al. (2016) reported a positive correlation only between *E. coli* and GI of HuNoV. There was no correlation between *E. coli* and GII HuNoV or HAV (Lopez-Galvez et al., 2016). (Horman et al. (2004) reported that a positive relation between *E. coli* and HuNoV in surface water, although it was a poor indicator for HuNoV in well water.

In our study, we did not find any correlation between detection levels for generic *E. coli* and that of HuNoV. Since generic *E. coli* exhibits high persistence in water, especially in tropical regions, its presence does not always indicate fecal contamination (Berthe et al., 2013). Generic *E. coli* persistence in water is variable based on matrix effect, antagonistic microflora and other factors. In addition, generic *E. coli* could be detected in both human and animal feces. Detected generic *E. coli* is likely sourced from animal feces, as some sampling sites in this study were surrounded by cattle ranches and/or exposed to wild animals and birds. Other markers have also been developed to estimate fecal contamination. FRNA bacteriophages have been employed as enteric viral indicator for fecal contamination in tropical aquatic systems (Arredondo-Hernandez et al., 2017) and host-specific *bacteroidale* markers have been employed as indicators for fecal contamination (Wilkes et al., 2013). With the improved purification and RT-qPCR methods, it might be more convenient to measure HuNoV directly from environmental water samples rather than using indirect indicators.

The Carr Lake (C), Tembladero Slough (T) and Lower Salinas River regions were regarded as human-impacted areas. Despite expectations of higher detection rates, we did not observe significant differences in HuNoV detection rates between human-impacted areas and animal-impacted areas. The discovery of high detection rates at upper Salinas River sites was surprising, as they are located far from human populations. However, it needs to be noted that wild animals, including wild pig and cattle, inhabit the area. Cheetham et al. (2006) reported that GII HuNoV could replicate in gnotobiotic pigs. It is possible that wild animals could serve as hosts for HuNoV and is the source of HuNoV contamination in this area, and this likelihood requires further study.

In summary, we demonstrated that high prevalence of HuNoV and bacterial pathogens were detected in environmental water samples in the region. Overall, there was no significant difference in HuNoV detection rates between samples testing positive or negative for the four bacterial pathogens tested. There were significant higher detection rates in animal-impacted areas than that of human-impacted areas for bacterial pathogens but not for HuNoV. The overall detection levels of generic *E. coli* and detection rate of HuNoV showed no correlation.

## Author Contributions

MC and LG designed experiments for bacterial pathogens. PT and BQ designed experiments for detection and genotyping of HuNoV. LS, DY, QL, DW, and BL carried out experiments for detection and genotyping of HuNoV.

## Conflict of Interest Statement

The authors declare that the research was conducted in the absence of any commercial or financial relationships that could be construed as a potential conflict of interest.
